# Advances in [^18^F]Trifluoromethylation Chemistry for PET Imaging

**DOI:** 10.3390/molecules26216478

**Published:** 2021-10-27

**Authors:** Felix Francis, Frank Wuest

**Affiliations:** 1 Department of Chemistry, University of Alberta, 11227 Saskatchewan Drive NW, Edmonton, AB T6G 2N4, Canada; ff2@ualberta.ca; 2 Department of Oncology, University of Alberta, 11560 University Avenue, Edmonton, AB T6G 1Z2, Canada

**Keywords:** fluorine-18, trifluoromethylation chemistry, positron emission tomography (PET)

## Abstract

Positron emission tomography (PET) is a preclinical and clinical imaging technique extensively used to study and visualize biological and physiological processes in vivo. Fluorine-18 (^18^F) is the most frequently used positron emitter for PET imaging due to its convenient 109.8 min half-life, high yield production on small biomedical cyclotrons, and well-established radiofluorination chemistry. The presence of fluorine atoms in many drugs opens new possibilities for developing radioligands labelled with fluorine-18. The trifluoromethyl group (CF_3_) represents a versatile structural motif in medicinal and pharmaceutical chemistry to design and synthesize drug molecules with favourable pharmacological properties. This fact also makes CF_3_ groups an exciting synthesis target from a PET tracer discovery perspective. Early attempts to synthesize [^18^F]CF_3_-containing radiotracers were mainly hampered by low radiochemical yields and additional challenges such as low radiochemical purity and molar activity. However, recent innovations in [^18^F]trifluoromethylation chemistry have significantly expanded the chemical toolbox to synthesize fluorine-18-labelled radiotracers. This review presents the development of significant [^18^F]trifluoromethylation chemistry strategies to apply [^18^F]CF_3_-containing radiotracers in preclinical and clinical PET imaging studies. The continuous growth of PET as a crucial functional imaging technique in biomedical and clinical research and the increasing number of CF_3_-containing drugs will be the primary drivers for developing novel [^18^F]trifluoromethylation chemistry strategies in the future.

## 1. Introduction

**Figure 1:**
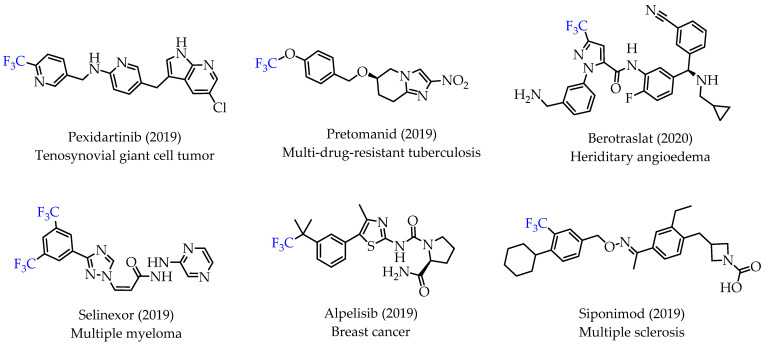
Representative examples of recently approved drugs with trifluoromethyl groups.

Trifluoromethyl (CF_3_) groups are important functional groups in pharmaceutical and medicinal chemistry as excellent bioisosteres of methyl groups. CF_3_ groups are incorporated into drugs to improve potency, binding selectivity, lipophilicity and facilitate their administration [1,2]. Improved pharmacological activities and properties are observed with numerous CF_3_ group-containing drugs. This trend is also visible in recent FDA approvals for drugs containing a CF_3_ group (Figure 1) [3].

Many small molecule drugs recently approved for cancer treatment have at least one fluorine atom, underlining the importance of fluorine atoms as effective bioisosteres. The installation of one or multiple fluorine atoms into small molecule compounds can significantly improve metabolic stability, increase bioavailability and cellular membrane permeability, thus resulting in drugs with better pharmacokinetics and enhanced efficacy [3,4,5,6,7].

The rapidly growing number of CF_3_-containing drugs has triggered novel synthetic methodologies for incorporating CF_3_-groups into small molecules. Trifluoromethylation reactions entail electrophilic, nucleophilic, and radical synthetic methods. Significant advances include better and more readily available CF_3_ group sources and optimizing new chemistry for site-specific CF_3_ group incorporation. The synthesis of CF_3_-containing compounds traditionally involves multi-step and long synthesis sequences utilizing various trifluoromethylated synthons. However, conventional CF_3_ chemistry has been replaced by more practical transition metal-catalysed trifluoromethylation reactions. The most widely used transition metal for trifluoromethylation reactions is Cu, along with Pd and Ni. 

**Figure 2:**
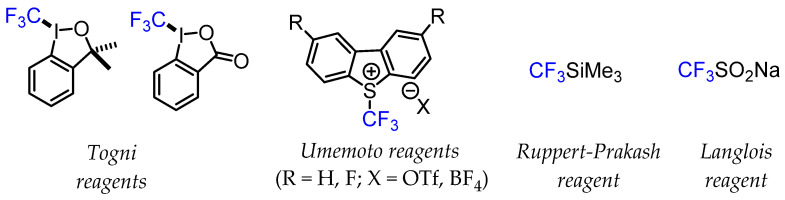
Examples of common trifluoromethylating reagents.

The development of various stable CF_3_-containing sulfinate salts such as Langlois’ reagent (CF_3_SO_2_Na) proved to be a flexible approach for trifluoromethylation reactions via a radical mechanism [8,9]. The emergence of difluoromethylated precursors for the rapid generation of CF_3_ groups is also an important strategy. Nucleophilic Ruppert–Prakash reagent (CF_3_SiMe_3_) and similar silyl reagents have also been integral in popularising trifluoromethylation reactions. The development of hypervalent iodine compounds (Togni reagents) and sulfonium salts (Umemoto reagents) for electrophilic CF_3_ group transfer has also significantly extended the possibilities of trifluoromethylation chemistry [10]. These nucleophilic, electrophilic, and radical CF_3_ transfer reagents were tested under different experimental conditions, such as standalone reagents for direct trifluoromethylation reactions or as part of transition metal-mediated methods for CF_3_ group transfer chemistry (Figure 2).

The rapidly increasing number of novel trifluoromethylation chemistry techniques and their impact on drug development and agrochemistry were summarised in detail in several recent review articles [1,2,4,7,10,11,12,13]. In this review, we present recent results of significant [^18^F]trifluoromethylation chemistry strategies for using [^18^F]CF_3_-containing radiotracers in preclinical and clinical positron emission tomography (PET) imaging studies. The continuous growth of PET as a functional imaging technique in biomedical and clinical research and the increasing number of CF_3_-containing drug compounds are the primary drivers for developing novel [^18^F]trifluoromethylation chemistry strategies.

## 2. PET Imaging and Fluorine-18

PET is an important molecular imaging technique with unique diagnostic applications for the quantitative assessment of biochemical and physiological processes in vivo with specific radiotracers. PET is also used to assess the pharmacokinetics and pharmacodynamics of novel drug candidates [14,15,16,17,18,19]. As a high sensitivity imaging technique, a typical PET experiment uses radiotracer doses in the nanomolar to picomolar concentration range. Such low administered doses generally prevent pharmacological and toxicological effects making PET studies a safe diagnostic procedure in the clinic. 

**Figure 3:**
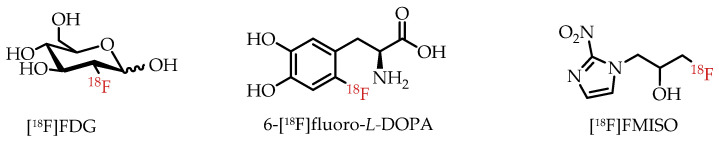
Prominent examples of clinically used fluorine-18-labelled PET radiotracers.

Fluorine-18 (^18^F) is among the most commonly used radioisotopes for PET, as demonstrated by hundreds of fluorine-18-labelled radiotracers [16,17]. Prominent examples of clinically used PET radiotracers are depicted in Figure 3. The most widely and frequently used PET radiotracer by far is glucose analogue 2-[^18^F]fluoro-2-deoxy-D-glucose ([^18^F]FDG). [^18^F]FDG is particularly useful in measuring enhanced glucose metabolism due to altered energy metabolism, also referred to as the Warburg effect, in many cancers [20]. 1*H*-1-(3-[^18^F]Fluoro-2-hydroxypropyl)-2-nitroimidazole ([^18^F]FMISO) is another example of a fluorine-18-labelled radiotracer used in the clinic for the visualization of tissue or organ hypoxia in cancer and other diseases [21]. The importance of ^18^F-labeled radiotracers for clinical PET was recently demonstrated by the FDA approval of fluorine-18-labelled amino acid [^18^F]Fdopa for clinical PET imaging of Parkinson’s disease [3]. 

Many organofluorine compounds and the available pharmacological data of trifluoromethylated drugs place CF_3_ groups among the most predictable functional groups in medicinal chemistry and drug development. The high popularity of using CF_3_ groups in many drug molecules is also interesting for developing radiotracers labelled with the short-lived positron emitter fluorine-18. Replacing one of the three fluorine atoms in a CF_3_ group of a given compound with a fluorine-18 atom would provide a versatile and broadly applicable class of radiotracers for PET imaging. Although some radiochemistry strategies have been reported for this conversion, they are far from optimal [22]. Compared to other commonly employed fluorine-18 radiochemistry methods such as aliphatic and aromatic nucleophilic radiofluorinations, [^18^F]trifluoromethylations are still relatively underexplored. 

### PET and [^18^F]CF_3_ Group Chemistry

The development of fluorine-18-labelled PET imaging agents benefits significantly from the availability of numerous marketed fluorinated drugs and drug analogues. The existing large number of fluorine-containing drugs and the available literature on their pharmacokinetics/pharmacodynamics and toxicological profile provide an excellent starting point for the design and synthesis of respective fluorine-18-labelled compounds. Among the plethora of fluorine-containing drugs, compounds with CF_3_ groups represent exciting leads for developing fluorine-18 radiotracers.

**Figure 4:**
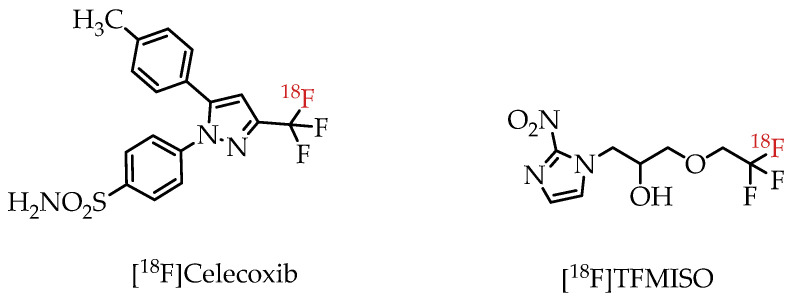
[^18^F]Trifluoromethylated PET imaging agents [^18^F]Celecoxib and [^18^F]TFMISO.

There have already been several examples utilizing trifluoromethylated drugs as PET probes. Prominent examples include fluorine-18-labelled COX-2 inhibitor [^18^F]Celecoxib [23] and hypoxia imaging agent [^18^F]TFMISO (Figure 4) [24]. 

However, a particular challenge in synthesizing [^18^F]trifluoromethylated compounds is the typically obtained relatively low molar activities of the radiotracers. This is mainly due to undesired ^18^F-^19^F isotopic exchange reactions contributing to the radioactive product and lowering molar activity. Therefore, the development of novel [^18^F]trifluoromethylation methods primarily aims to increase molar activities of the final radiotracer while achieving reasonable radiochemical yields. 

## 3. Major Strategies for the Generation of [^18^F]Trifluoromethyl Groups

**Figure 5:**
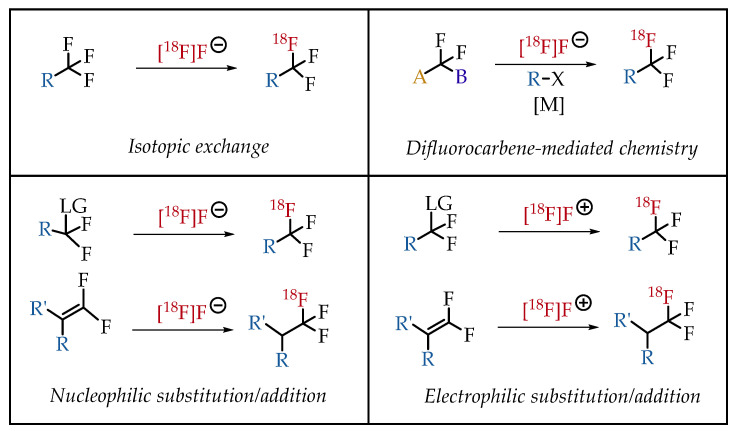
General synthesis strategies for the generation of [^18^F]CF_3_ groups.

In general, there are four strategies to synthesize [^18^F] CF_3_ groups (Figure 5).

### 3.1. ^19^F-to-^18^F Isotopic Exchange Chemistry

^19^F/^18^F isotopic exchange reactions have many advantages over other strategies, mainly the ease of substrate availability. The commercial availability of many drugs and substrates containing CF_3_ groups makes this strategy a versatile synthesis approach with broad operational flexibility. However, the selective C-F bond cleavage represents a challenge as the harsh conditions required for C-F bond cleavage significantly reduces functional group tolerance. Late-stage radiolabelling strategies are also relatively underdeveloped. Despite several challenges and limitations, isotopic exchange protocols have been frequently used for the radiolabelling CF_3_ groups in the past, mainly due to the surprising tolerance to the presence of water [22]. The final radiolabelled compound and the starting compound would be chemically the same and pose a critical risk of diluting molar activity.

### 3.2. Nucleophilic Substitution/Addition Radiofluorinations

Nucleophilic substitution/addition radiofluorination chemistry is among the most popular radiochemistry strategies for synthesizing [^18^F]CF_3_ groups, as exemplified by numerous published synthesis protocols. The chemistry stems from the extensive experience and success with nucleophilic radiofluorinations, including nucleophilic addition of [^18^F]fluoride across electron-deficient alkenes. Consequently, nucleophilic substitution/addition radiofluorination chemistry was explored more extensively than other strategies to synthesize [^18^F]CF_3_ groups. This success of this route could be because of the least probability of intrinsic molar activity dilution. The most challenging part of this chemistry is the synthesis of precursors that can undergo a facile nucleophilic substitution. 

### 3.3. Difluorocarbene-Mediated Chemistry 

**Figure 6:**
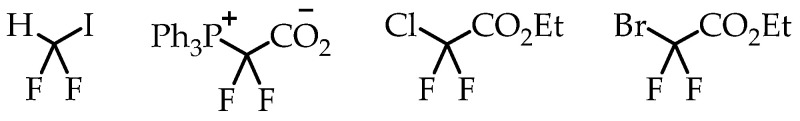
Difluororcarbene precursors.

Difluorocarbene-mediated chemistry is also a popular synthesis route for [^18^F]CF_3_ group generation. The simplicity in reaction design and availability of difluorocarbene sources helped to make this synthesis strategy a popular method for developing [^18^F]CF_3_-containing radiotracers. Multiple difluorocarbene sources have been identified and utilized with varying success (Figure 6) [25,26,27]. This route has been optimized for a wide variety of substrates such as aryl halides and sulfhydryl groups. The possibility of fluoride scrambling is a threat to molar activity. However, various reports have shown that this method is capable of [^18^F]trifluoromethylation.

### 3.4. Electrophilic Radiofluorinations

Electrophilic fluorination reactions are the dominant synthesis method of generating organofluorine compounds. Electrophilic fluorinations are accomplished via direct fluorination with fluorine gas or by electrophilic fluorinating reagents such as Selectfluor (1-chloromethyl-4-fluoro-1,4-diazoniabicyclo[2.2.2]octane bis(tetrafluoroborate)). In the case of trifluoromethylation reactions, electrophilic fluorination chemistry holds several advantages. Hydrated [^18^F]fluoride is mostly ineffective in nucleophilic attacks; its use in aqueous and other protic solvents is cumbersome. In the case of fluorine-18 radiochemistry, electrophilic fluorine-18 transferring reagents such as [^18^F]Selectfluor step in to fill that gap. However, only little research has been reported to use electrophilic fluorine-18 transfer reagents to synthesise [^18^F]CF_3_ groups. Typical electrophilic radiofluorinating agents include [^18^F]Selectfluor, [^18^F]F_2_ and [^18^F]trifluoromethylated Umemoto reagents [28,29,30,31]. A major concern is the low molar activity associated with this method. Most of the electrophilic syntheses start with carrier-added fluorine-18, which severely dilutes the molar activity.

## 4. Examples of [^18^F]CF_3_ Compounds

### 4.1. Aliphatic [^18^F]CF_3_ Compounds

Dolbier et al. reported the electrophilic radiofluorination of a 2,3,3-trifluoroallyl group to synthesize hypoxia marker EF5 [32]. This process yielded 17% of EF5.

**Scheme 1:**
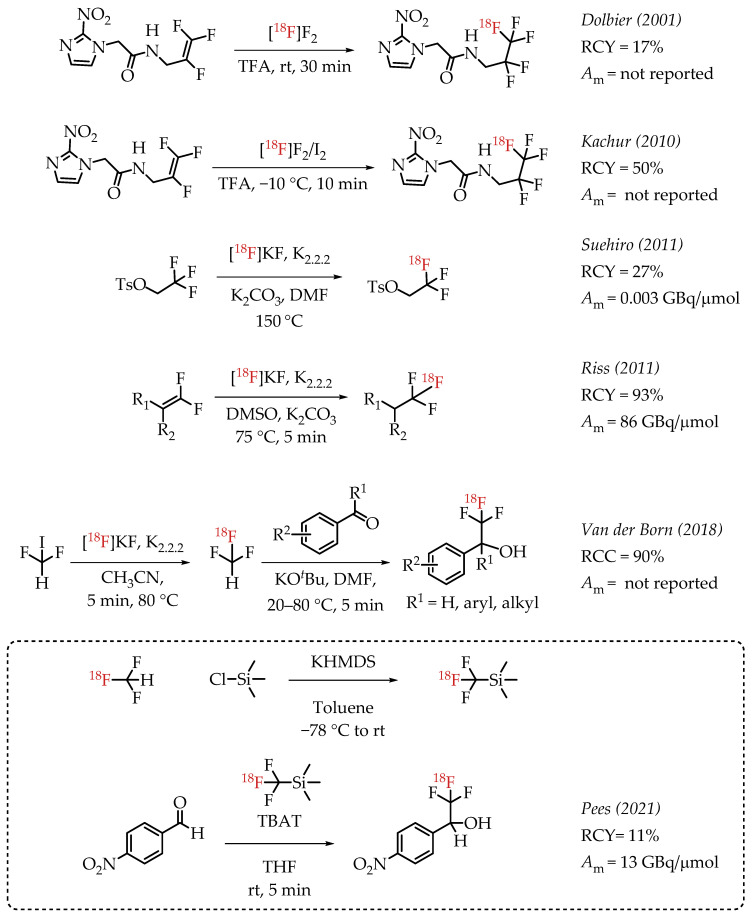
Examples of aliphatic [^18^F]CF_3_ compounds. (RCY: radiochemical yield; RCC: radiochemical conversion; A_m_: molar activity.)

In 2010, Kachur et al. reported an improved strategy for the synthesis of EF5 [33]. The study added catalytic amounts of iodine, and the authors observed that 1% iodine improved the radiochemical yields to 50%. Suehiro et al. employed an ^18^F/^19^F isotopic exchange for the synthesis of [^18^F]CF_3_ groups as part of the synthesis of hypoxia imaging agent [^18^F]TFMISO [27]. The described procedure generated [^18^F]TFMISO in reasonable radiochemical yields (60%) but at a low molar activity (0.003 GBq/μmol). Riss et al. reported a nucleophilic alkene [^18^F]fluorination method resulting in products at a high molar activity (86 GBq/μmol) [34]. The method was limited to the generation of 2,2,2-[^18^F]trifluoroethyl groups, but the operational simplicity of the chemistry and the obtained high molar activities are remarkable. While the molar activity of the method is impressive, the radiochemical yield was low [35]. Van der Boon et al. published a protocol using difluoroiodomethane as a building block to generate [^18^F]CF_3_ groups. The process used a nucleophilic aliphatic radiofluorination reaction of no-carrier-added (n.c.a.) [^18^F]fluoride with difluoroiodomethane to form [^18^F]fluoroform. [^18^F]fluoroform was deprotonated and added to an aldehyde yielding respective [^18^F]trifluoromethyl carbinols in good yields. The experimental design of the reaction was relatively complex as it involves low-temperature distillation and trapping of highly volatile [^18^F]fluoroform at −80 °C in a secondary reaction vessel [22,36]. In 2021, Pees and co-workers reported the synthesis of [^18^F]Ruppert-Prakash reagent ([^18^F]Trifluoromethyltrimethylsilane) [37]. The synthesis of [^18^F]Ruppert-Prakash reagent was achieved by the deprotonation of [^18^F]fluoroform with KHMDS and subsequent addition of this nucleophile to trimethylsilyl chloride to generate the [^18^F]trifluoromethyl transfer reagent. The synthetic utility of this method was demonstrated by the reaction of [^18^F]Ruppert-Prakash reagent with aldehydes, where TBAT (Tetrabutylammonium difluorotriphenylsilicate) was used as the initiator. The reaction with 4-nitrobenzaldehyde generated the radiolabelled compound in 11% RCY and 13 GBq/μmol molar activity (Scheme 1).

Prakash et al. demonstrated the radiolabelling of protected enols as a strategy for synthesizing [^18^F]CF_3_ groups. However, the method was limited to the synthesis of [^18^F]trifluoroacetoaryl ketones [38]. The strategy utilized electrophilic radiofluorination with [^18^F]F_2_ resulting in products with diminished molar activity in the range of 0.02 GBq/μmol. Gomez et al. reported a method for the synthesis of fluorine-18-labelled trifluoroacetamides [39]. The direct nucleophilic displacement of difluorobromides with [^18^F]fluoride was not efficient. The addition of metal salts was also not successful, whereas the addition of base DBU proved to be crucial, and radiochemical conversion yields of 71% were observed. Further exploration of other nitrogen-based nucleophilic activators such as DBU confirmed the beneficial effects of nitrogen bases. The use of guanidine derivatives TBD and MTBD further improved the radiochemical conversions to 81% and 76%, respectively. The authors reported a moderate molar activity of 8.4 GBq/μmol for the reaction. Meyer and co-workers reported a nucleophilic halex reaction to synthesize α-CF_3_ ketones [40]. The reaction used TBD (1,5,7-triazabicyclo-[4.4.0]dec-5-ene) as additive. They were able to obtain a 30% yield and a molar activity up to 1.27 GBq/μmol.

**Scheme 2:**
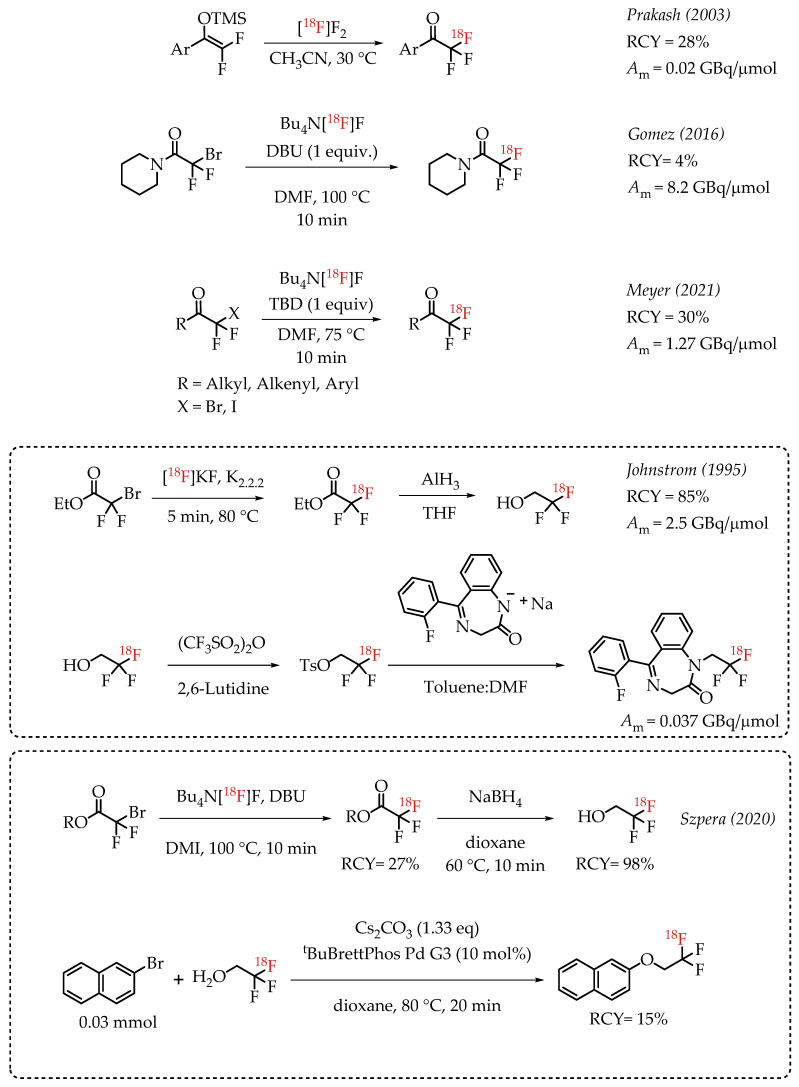
[^18^F]trifluoromethylation of α-trifluoromethyl carbonyls. (RCY: radiochemical yield; A_m_: molar activity).

Johnstrom et al. reported the formation of ethyl [^18^F]trifluoroacetate during the synthesis of 2,2,2-[^18^F]trifluoroethyl triflate [41]. The authors subjected the [^18^F]CF_3_ compound to hydride reduction to obtain their target molecule. The final product (a fluorine-18-labelled 1,4-benzodiazepine-2-one) was obtained at a molar activity of 0.037 GBq/μmol. In 2020, Szpera and co-workers reported an improved synthesis of 2,2,2-[^18^F]trifluoroethanol [42]. The overall strategy of radiofluorination of α-diflurobromo ester to generate the radiolabelled ester and a further reduction of the ester to corresponding alcohol was similar to that of Johnstrom et al. The higher molar activity radiofluorination route disclosed by Gomez and co-workers was adapted to this synthesis, where DBU is used as an additive to facilitate the radiofluorination (Scheme 2).

**Scheme 3:**
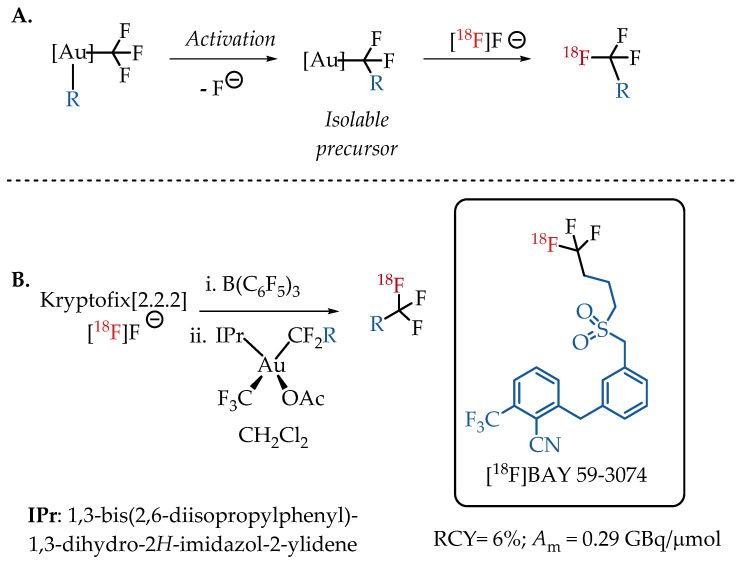
(**A**) Strategy of Levin et al. utilising Au(III)-CF_3_ complexes to facilitate the isotopic exchange with [^18^F]fluoride; (**B**) Radiolabelling of [^18^F]BAY 59-3074, a cannabinoid agonist. (RCY: radiochemical yield; A_m_: molar activity.)

Levin et al. demonstrated an alternative isotopic fluorine exchange method for [^18^F]CF_3_ labelling. Here, the CF_3_ group is coordinated to an Au(III) complex. The Au(III) complex facilitates the C-F bond cleavage and migratory insertion to generate an isolable R-CF_2_-metal complex, which in turn provides easy access to the [^18^F]CF_3_ moiety [43]. This unique protocol enables the preparation of fluorine-18-labelled aliphatic CF_3_-containing radiotracers in 6% radiochemical yield and with a low molar activity in the range of 0.3 GBq/μmol. The feasibility of using bis(trifluoromethyl)Au(III) complexes for the isotopic exchange with [^18^F]fluoride was successfully demonstrated with the synthesis of cannabinoid agonist [^18^F]BAY 59-3074 (Scheme 3).

**Scheme 4:**
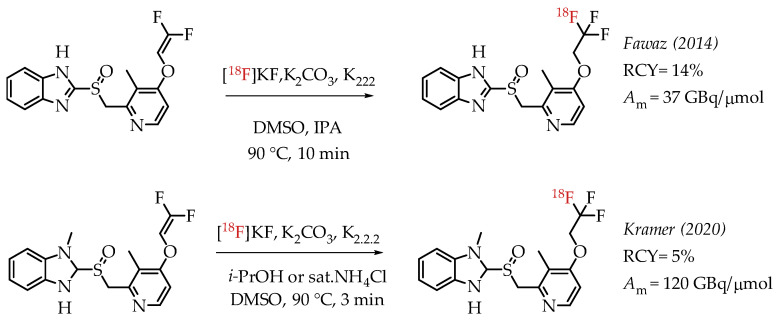
Radiolabelling of lanzoprazole ([^18^F]LNS) and *N*-methyl lanzoprazole ([^18^F]NML). (RCY: radiochemical yield; A_m_: molar activity.)

Fawaz et al. reported the synthesis of [^18^F]lanzoprazole ([^18^F]LNS) and its subsequent analysis as a potential radiotracer for quantifying aggregated tau protein levels in Alzheimer’s disease (AD) and progressive supramolecular palsy (PSP) [44]. The authors followed the nucleophilic addition chemistry route of n.c.a. [^18^F]fluoride on a difluoromethylidene to generate the [^18^F]CF_3_ group (Scheme 4). They observed the formation of a [^18^F]difluoromethylidene compound as the primary side product during the reaction. The combined radiochemical yield was 14%, including the side product. Further optimization did not improve the radiochemical yield. Moreover, the authors found that the ratio of [^18^F]difluoromethylidene increased, and the radiochemical yield of [^18^F]LNS was not higher than 7%. 

**Figure 7:**
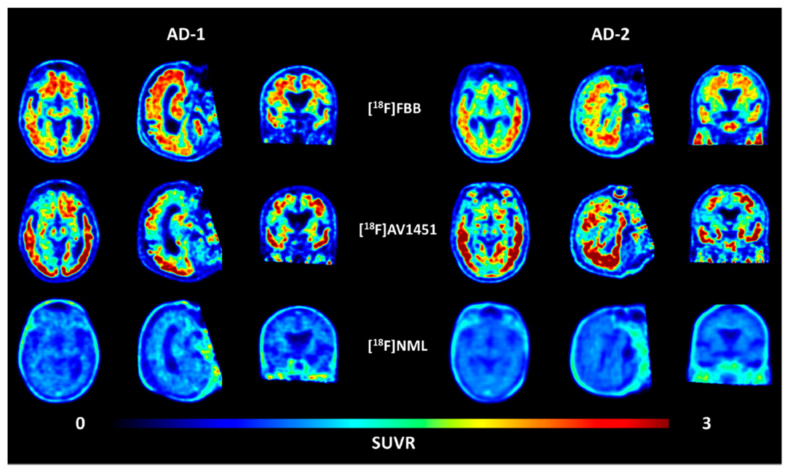
Transversal, sagittal, and coronal view of averaged PET images of [^18^F]AV1451 and [^18^F]NML uptake in two AD patients 90–110, 80–100, and 60–90 min p.i., respectively. (Images taken from reference [45]).

Kramer et al. described the synthesis of fluorine-18-labelled *N*-methyl lanosoprazole ([^18^F]NML), an analogue of lansoprazole (Scheme 4) [45]. They followed the procedure reported by Fawaz et al. involving nucleophilic addition of n.c.a. [^18^F]fluoride to the corresponding difluoro-enol ether labelling precursor [44]. This process afforded the radiotracer in radiochemical yields of 4.6% at high molar activities of 120 GBq/μmol. In the clinic, radiotracer [^18^F]NML showed good brain uptake and favourable pharmacokinetics, but the brain retention in AD and PSP patients was low (Figure 7).

**Scheme 5:**
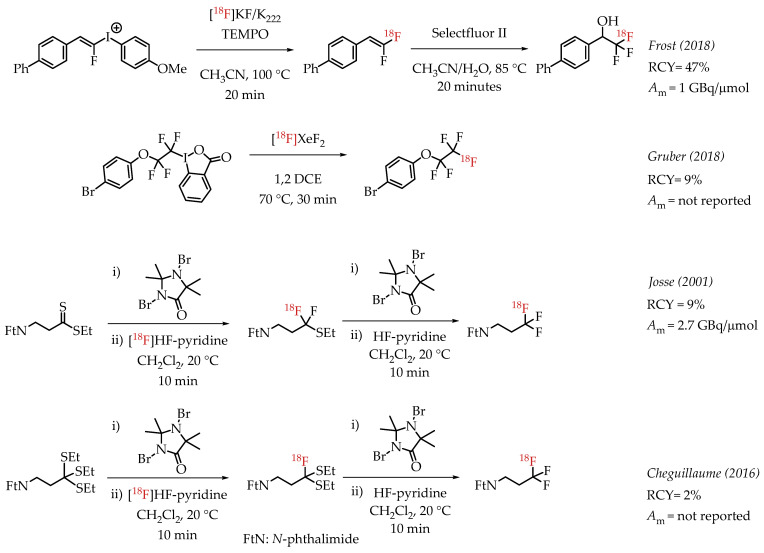
Additional examples of aliphatic [^18^F]CF_3_ compounds. (RCY: radiochemical yield; A_m_: molar activity.)

Additional examples of aliphatic [^18^F]CF_3_ compounds are summarized in Scheme 5. Frost et al. described the synthesis of 1,1-[^18^F]difluoroalkenes from the reaction of [^18^F]fluoride and fluoroalkenyl(4-methoxyphenyl)iodonium triflates [46]. The nucleophilic substitution on the iodonium salt generated the respective fluorine-18-labelled difluoroalkene, and subsequent fluorination of the alkene with Selectfluor II yielded the [^18^F]CF_3_ group.

Gruber et al. discussed the synthesis of [^18^F]pentafluoroethyl groups from hypervalent iodanes via an electrophilic radiofluorination reaction with [^18^F]XeF_2_ [47]. This was as part of a more extensive study involving electrophilic substitution reactions of iodanes with different electrophiles, including [^18^F]XeF_2_. The reaction of [^18^F]XeF_2_ with a cyclic perfluorinated iodane afforded a [^18^F]CF_3_CF_2_-containing compound with a radiochemical yield of 9%. A carrier-added radiosynthesis reported by Josse et al. achieved a molar activity of 2.7 GBq/μmol [48]. The procedure involved a fluorodesulphurization of a dithioester, which had to be subjected to another desulphurization reaction to furnish the [^18^F]CF_3_ group. Cheguillaume et al. reported a strategy for synthesizing perfluorinated alkyl motifs to develop novel nitroimidazole-based hypoxia PET markers [49]. In this report, the authors utilized a 1,3-dibromo-5,5-dimethylhydantoin (DBH)-activated radiofluorination of trithioorthoesters with [^18^F]HF-pyridine followed by an additional DBH-activated fluorination step with an excess of HF-pyridine to yield [^18^F]CF_3_-containing compounds.

### 4.2. Aromatic [^18^F]CF_3_ Compounds

**Scheme 6:**
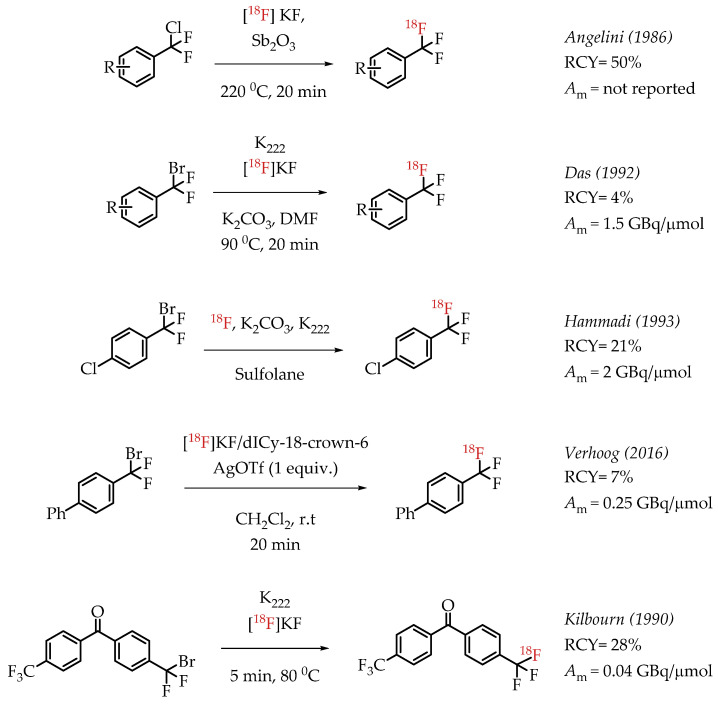
Examples of aromatic [^18^F]CF_3_ compounds. (RCY: radiochemical yield; A_m_: molar activity.)

Angelini et al. attempted the synthesis of [^18^F]CF_3_ groups by a Lewis acid-mediated dechlororadiofluorination using [^18^F]fluoride and Sb_2_O_3_ [50]_._ This study resulted in the generation of [^18^F]CF_3_ groups at low molar activities and moderate radiochemical yields. Das et al. utilized the high reactivity of the benzylic position in various bromo-difluoromethyl aryl compounds for nucleophilic substitution reactions with n.c.a. [^18^F]fluoride. The resulting [^18^F]CF_3_-containing compounds were obtained in low radiochemical yields of 2–4% at a low molar activity (1.5 GBq/μmol) [51]. Authors observed an inverse correlation between the obtained molar activity and the reaction temperature. Verhoog et al. discussed a [^18^F]trifluoromethylation reaction via Ag(I)-mediated halogen exchange with aryl difluorobromides [52]. The reaction can be performed at room temperature at radiochemical yields of 7% and molar activities of 0.25 GBq/μmol. Kilbourn et al. reported the synthesis of aryl [^18^F]CF_3_ groups with a molar activity of 0.04 GBq/μmol. The examples of these halex reactions are summarized in Scheme 6.

**Scheme 7:**
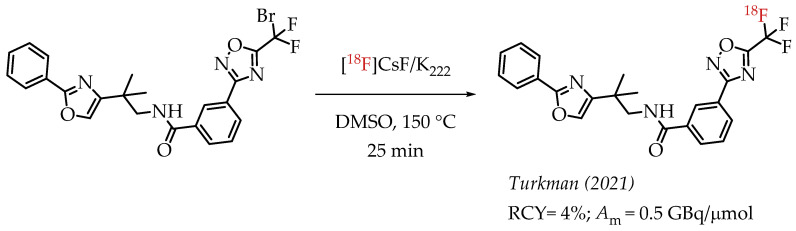
Turkman’s synthesis of [^18^F]TMP195. (RCY: radiochemical yield; A_m_: molar activity.)

**Figure 8:**
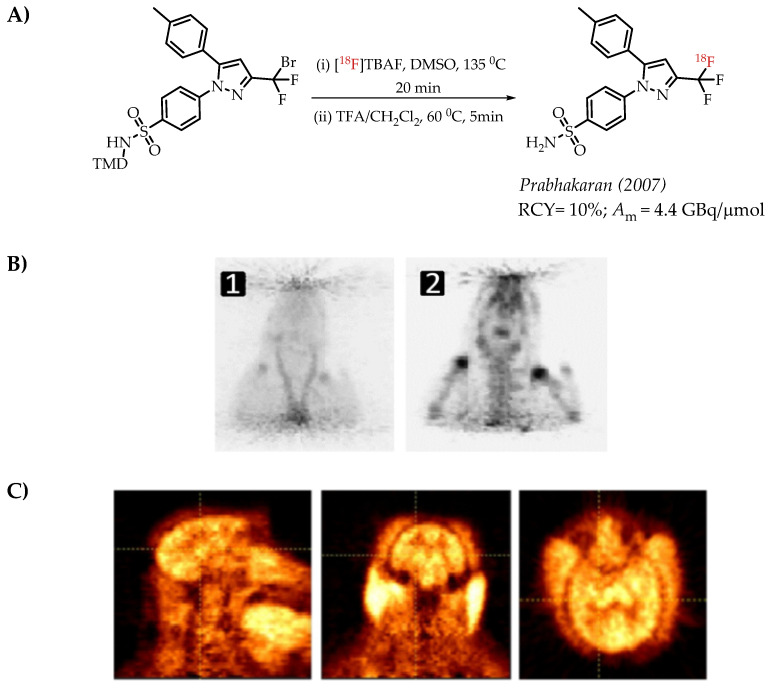
(**A**) Radiolabelling of celecoxib reported by Prabhakaran et al., (**B**) microPET images of [^18^F] celecoxib in a rat. PET images of [^18^F] celecoxib in a rat (images are normalized to the injected dose). (1) Sum of 0–20 min PET images; (2) sum of 60–120 min PET images (**C**) Sum of the (0–120 min) PET images of a baboon brain after the injection of 44 MBq of [^18^F]Celecoxib. First column, sagittal; middle column, coronal; last column, axial views. (Adopted from [23]; RCY: radiochemical yield; A_m_: molar activity).

The authors used a direct nucleophilic radiofluorination reaction with n.c.a. [^18^F]fluoride on difluoroarylbromide to yield the desired [^18^F]CF_3_ compound [53]. The described [^18^F]CF_3_ chemistry was part of a multi-step synthesis of a fluorine-18-labelled GABA uptake inhibitor. Hammadi et al. reported the nucleophilic displacement of a benzylic bromide to generate 4-chloro-α,α,α-[^18^F]trifluoromethyl toluene in moderate radiochemical yields (~30%) but low molar activities in the range of 1 GBq/μmol [54]. The fluorine-18-labelled aryl chloride was further used for the synthesis of selective serotonin uptake inhibitor [^18^F]-(*S*)-fluoxetine. The authors observed that achieving higher molar activity depends on a delicate balance between temperature and precursor concentration. Prabhakaran et al. described the synthesis of [^18^F]celecoxib via direct nucleophilic substitution of the corresponding bromide [23]. The reaction provided radiotracer [^18^F]celecoxib at moderate molar activities of 4.5 GBq/μmol, which was tested for PET imaging in baboons and rats (Figure 8). A competitive [^19^F] to [^18^F] exchange reaction with the [^18^F] incorporation was a potential reason for the lower than anticipated molar activity. Turkman et al. published a nucleophilic radiolabelling of [^18^F]TMP195, a 5-[^18^F]-trifluoromethyl-1,2,4-oxadiazole (TFMO) containing radiotracer [55]. They synthesized the radiotracer with a 4% radiochemical yield and a relatively low molar activity of 0.5 GBq/μmol (Scheme 7). For this experiment, the [^18^F]fluoride was purchased from an external source, and it underwent significant decay (>2 half-lives) before the experiment. Authors hypothesized this as a reason for the overall low molar activity.

**Scheme 8:**
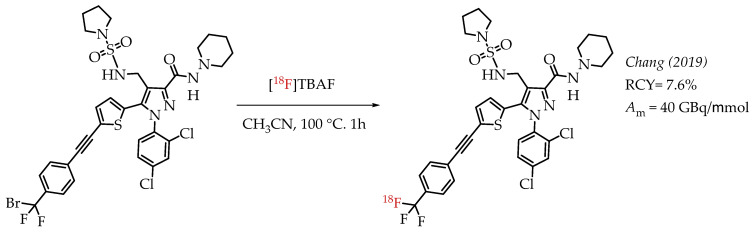
Radiosynthesis of [^18^F]DBPR211. (RCY: radiochemical yield; A_m_: molar activity.)

A peripheral cannabinoid receptor-1 (CB-1R) specific antagonist was labelled with ^18^F and tested in PET imaging experiments (Scheme 8). The study by Chang et al. demonstrated that radioligand [^18^F]DBPR211 showed only low blood-brain barrier penetration capacity in mice during the PET imaging experiments [56]. The radiolabelling precursor contained an aryl difluorobromomethyl group which has already been established as a suitable group for nucleophilic substitutions with n.c.a. [^18^F]fluoride to generate aryl [^18^F]CF_3_ groups. The radiolabelling was carried out in the presence of two unprotected N-H bonds, and the final product was isolated in radiochemical yields of 7.6% at a molar activity of 40 GBq/μmol.

**Scheme 9:**
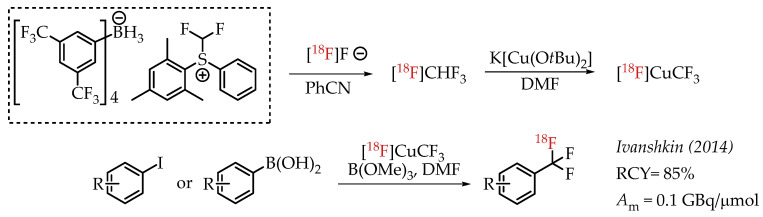
Difluormethylsulfonium salt-mediated radiolabelling strategy by Ivashkin et al. (RCY: radiochemical yield; A_m_: molar activity.)

Ivashkin et al. described a novel approach for the generation of [^18^F]CF_3_ groups (Scheme 9). The authors used [^18^F]fluoroform which was prepared from a difluormethylsulfonium salt [57]. [^18^F]fluoroform was converted into organometallic reagent [^18^F]CuCF_3_ using K[Cu(O*^t^*Bu)_2_], which in turn facilitates the cross-coupling reaction with aryl iodides or aryl boronic acids.

**Scheme 10:**
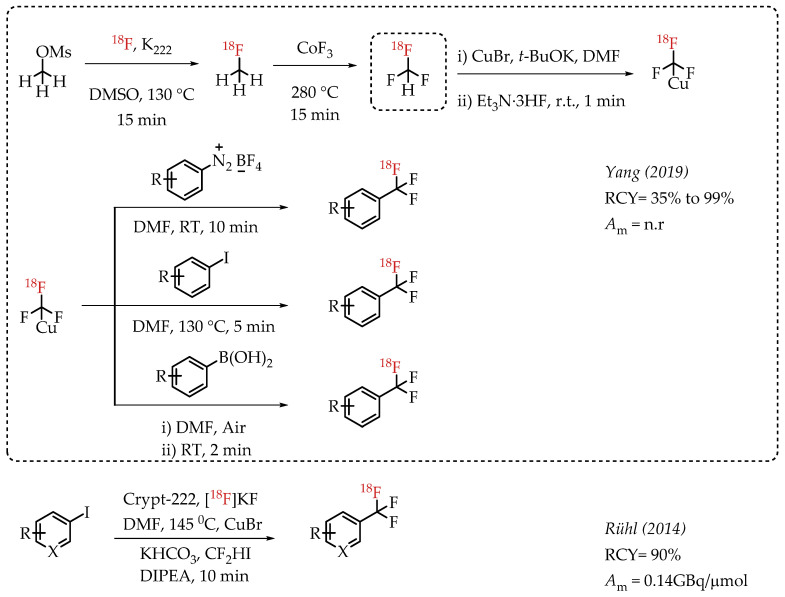
Gas-phase synthesis of [^18^F]fluoromethane and [^18^F]fluoroform by Yang et al.; Radiolabelling with Cu-[^18^F]trifluoromethyl complexes. (RCY: radiochemical yield; A_m_: molar activity).

Yang et al. reported a gas-phase synthesis route for the preparation of [^18^F]fluoroform via a two-step strategy involving [^18^F]fluoromethane (Scheme 10) [58]. [^18^F]fluoromethane synthesis was accomplished from methyl mesylate and n.c.a. [^18^F]fluoride. [^18^F]fluoromethane was further reacted with CoF_3_ to yield [^18^F]fluoroform in high molar activities of up to 163 GBq/μmol, averaging 36 GBq/μmol. The authors utilized [^18^F]fluoroform for an array of [^18^F]trifluoromethylations. Copper-mediated [^18^F]trifluoromethylation reactions with iodoarenes, aryl boronic acids and aryldiazonium salts afforded respective aromatic [^18^F]CF_3_ compounds in excellent radiochemical yields of up to 97%.

Rühl et al. utilized difluoroiodomethane as the difluorocarbene precursor. As a gas, difluoroiodomethane posed some inherent operational challenges. This chemistry converts CF_2_HI in the presence of CuBr into Cu-CF_3_ to improve the handling. In the presence of DIPEA and n.c.a. [^18^F]fluoride, reaction of Cu-CF_3_ with various iodoarenes afforded respective [^18^F]CF_3_-substituted aromatic compounds [59]. The chemistry reduces the dependence on strong bases to deprotonate CF_2_HI.

**Scheme 11:**
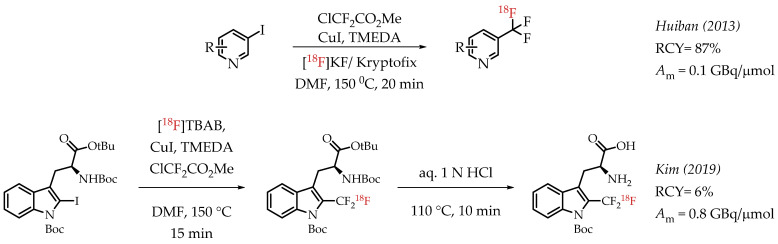
Cu mediated [^18^F]trifluoromethylation of aryl iodides. (RCY: radiochemical yield; A_m_: molar activity.)

**Figure 9:**
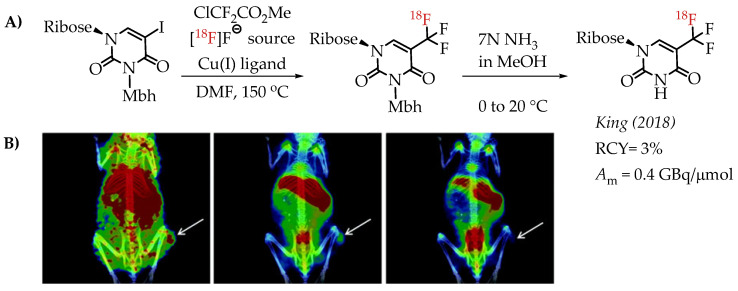
(**A**) Radiolabelling of trifluridine by King et al. (**B**) Representative PET/CT images selected from a 90 min dynamic PET scan of a mouse bearing an HCT116-derived tumour, following the administration of [^18^F]trifluridine. The white arrows indicate the tumour (adopted from [61]; RCY: radiochemical yield; A_m_: molar activity).

Huiban et al. discussed difluorocarbene-mediated chemistry with iodoarenes for the synthesis of aromatic [^18^F]CF_3_ compounds, however, at a low molar activity (0.1 GBq/μmol) [25]. The chemistry can be performed under open-flask conditions, highlighting the air stability and subsequent operational simplification. Substrates with unprotected carboxyl and hydroxyl groups gave only low radiochemical yields (>5%). In 2019 Kim et al. reported the synthesis of [^18^F]trifluoromethyl-l-tryptophan using Cu(I)-mediated [^18^F]trifluoromethylation to image the serotonergic system [60]. The Cu-difluorocarbene-mediated radiolabelling proceeded with a 6% radiochemical yield and a low molar activity of up to 0.76 GBq/μmol (Scheme 11). King et al. developed a method for the labelling of 5-trifluoromethyl-2-deoxyuridine (trifluridine) with fluorine-18 [61]. The difluorocarbene-mediated reaction provided [^18^F]trifluridine in radiochemical yields of 3% and a molar activity of 0.4 GBq/μmol. Radiotracer [^18^F]trifluridine was used for subsequent PET imaging describing the biodistribution and clearance profile of the compound in a tumour-bearing mouse (Figure 9).

Fu et al. reported a [^18^F]trifluoromethylation reaction for the synthesis of [^18^F]PTTP, a radiotracer that targets purinergic receptor P2X7 (P2X7R). P2X7R is an emerging molecular target for tracking and visualizing inflammation [62].

**Figure 10:**
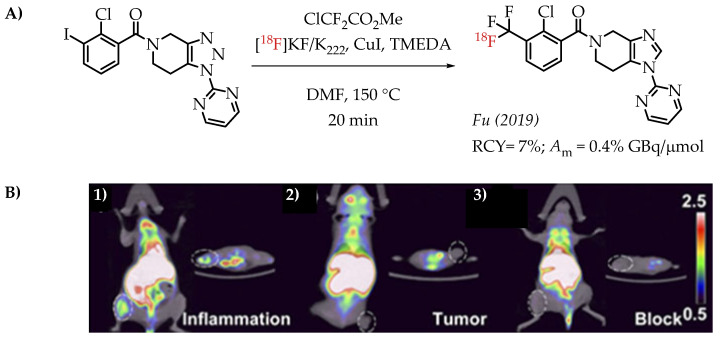
(**A**) Radiosynthesis of [^18^F]PTTP by Fu et al. (**B**) Small-animal PET/CT imaging of [^18^F]PTTP in mice: representative inflammation cohort (i), A549 tumour cohort (ii), and competitively blocked cohort (iii) at 5 min after injection (adopted from [62]; RCY: radiochemical yield; A_m_: molar activity).

The radiosynthesis involved a Cu-difluorocarbene-mediated reaction with an aryl iodide. ClCF_2_CO_2_Me was used as the difluorocarbene source. The radiosynthesis afforded [^18^F]PTTP in 7% radiochemical yield and a molar activity of 0.35 GBq/μmol suitable for PET imaging (Figure 10).

**Scheme 12:**
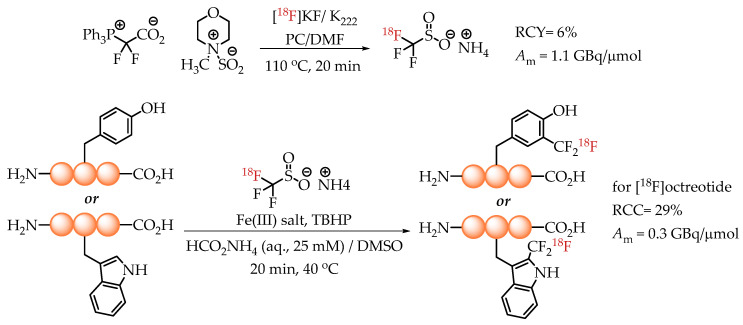
Strategy of Kee et al. for the radiolabelling of Tyr and Trp residues in peptides. (RCC: radiochemical conversion; A_m_: molar activity.)

In 2020, Kee et al. published an elegant method for the radiolabelling of unprotected native aromatic residues in peptides [26]. The novelty of this method lies in the generation of [^18^F]trifluoromethane sulfinate, an efficient and proven trifluoromethylating agent. The reaction was conducted via a multi-component assembly involving a difluorocarbene source (PDFA), [^18^F]fluoride and an SO_2_ source. The ammonium salt of [^18^F]trifluoromethane sulfinate was used for radiolabelling with n.c.a. [^18^F]fluoride in the presence of a Fe(III) salt and TBHP as a stoichiometric oxidant. The chemistry tolerates water, which enables its direct use for the radiolabelling of peptides under aqueous conditions. The described method was particularly effective for the introduction of [^18^F]CF_3_ groups into tyrosine and tryptophan residues of the peptide backbone. A successful application of the methodology yielded octreotide[Trp(2-CF_2_^18^F)] with a molar activity of 0.3 GBq/μmol (Scheme 12).

**Scheme 13:**
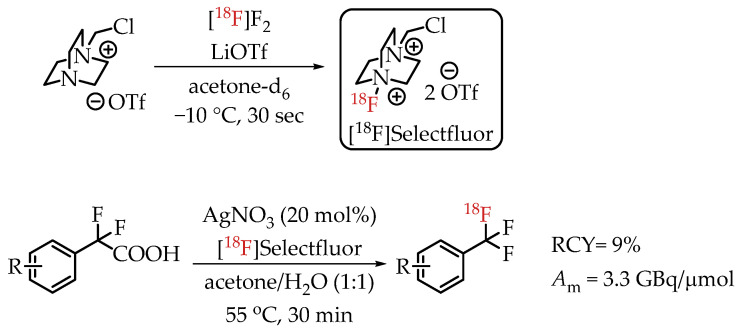
Decarboxylative electrophilic [^18^F]trifluoromethylation by Mizuta et al. (RCY: radiochemical yield; A_m_: molar activity.)

In 2013, Mizuta et al. published an Ag-catalysed decarboxylative radiofluorination with [^18^F]Selectfluor (Scheme 13) [31]. Electrophilic radiofluorinations using Selectfluor are stable towards water without the risk of inactivation. This carrier-added radiofluorination process yielded the target compounds in radiochemical yields greater than 95% and molar activities of 3.3 GBq/μmol.

**Scheme 14:**
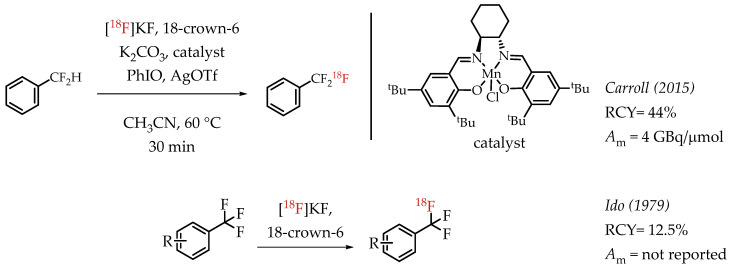
Additional examples of [^18^F]trifluoromethylation. (RCY: radiochemical yield; A_m_: molar activity).

Carroll et al. described an Mn-salen catalysed benzylic C-H activation for the radiofluorination of benzylic difluoromethanes [63]. The process uses a commercially available Jacobsen-type precatalyst and iodosobenzene as a stoichiometric oxidant. The authors reported good radiochemical yields and a molar activity in the range of 4 GBq/μmol. Among the earliest reports on the synthesis of [^18^F]CF_3_ compounds, Ido et al. demonstrated a fluorine isotopic exchange reaction to synthesize [^18^F]CF_3_ groups (Scheme 14) [22,64].

### 4.3. [18F]CF_3_-Heteroatom Compounds

**Scheme 15:**
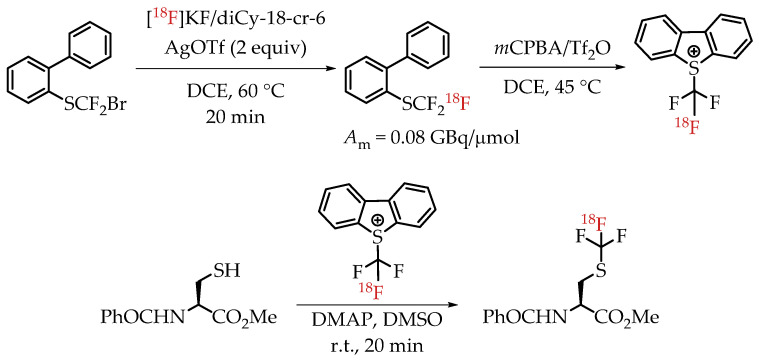
Radiosynthesis of [^18^F]CF_3_-S groups via [^18^F]Umemoto reagents. (A_m_: molar activity.)

Verhoog et al. utilized [^18^F]CF_3_ Umemoto reagents to facilitate the electrophilic [^18^F]trifluormethylation of unmodified cysteine residues [30]. The group was successful in synthesizing and using the new reagent for the radiolabelling of a variety of cysteine residues with a wide range of radiochemical yields and molar activities. The installation of [^18^F]CF_3_ group was accomplished via a nucleophilic halogen exchange reaction on a bromodifluoromethyl arylsulfide (Scheme 15). The molar activity of the reagent pre-cyclization (2-(trifluoromethylthio) biphenyl) was 0.08 GBq/μmol. The only reported molar activity of the final products was of the cyclic peptide cRADfC([^18^F]CF_3_), 0.15 GBq/μmol with a radiochemical yield of 20%.

**Scheme 16:**
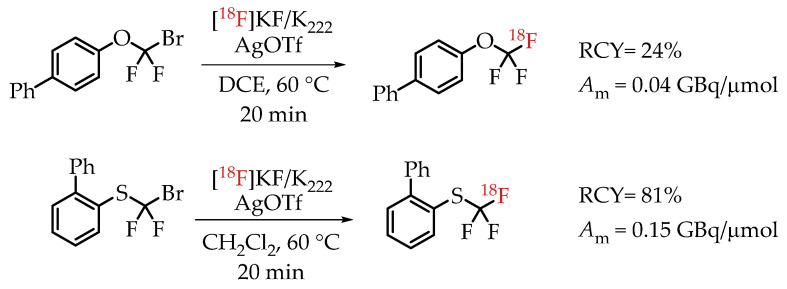
Synthesis of [^18^F]CF_3_-S and [^18^F]CF_3_-O groups reported by Khotavivattana et al. (RCY: radiochemical yield; A_m_: molar activity.)

[^18^F]Trifluoromethylation reactions with thiophenols and phenols were reported by Khotavivattana et al. [65]. The reaction proceeded via nucleophilic substitution with n.c.a. [^18^F]fluoride on bromodifluoromethylphenylsulfide and bromodifluoromethoxybenzene, respectively. The reaction required highly active AgOTf. The authors reported a significant reduction in the reactivity of AgOTf that was stored at room temperature and performed the addition of AgOTf inside a glovebox. The process yielded [^18^F]trifluoromethylated arenes with low molar activities in the range of 0.5 GBq/μmol (Scheme 16).

**Scheme 17:**
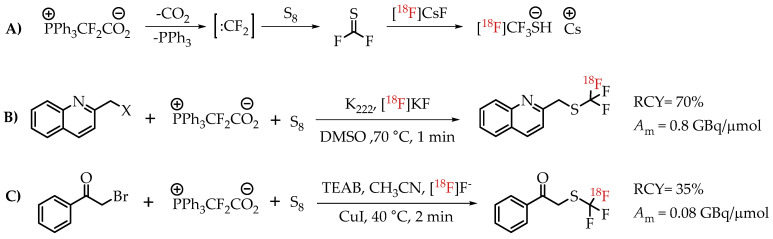
(**A**) Mechanism for the formation of [^18^F]CF_3_-S groups; (**B**) [^18^F]trifluoromethylthiolation of benzylic halides; (**C**) Cu-mediated [^18^F]trifluoromethylthiolation reaction of α-bromo carbonyls. (RCY: radiochemical yield; A_m_: molar activity.)

Liang et al. discussed an elegant synthesis route to access [^18^F]CF_3_-S groups employing novel reagent difluoromethylene phosphobetaine (PDFA) [27]. The described method opened the path for trifluoromethylthiolation reactions starting from several commercially available alkyl halides (Scheme 17).

Liang group followed up with a modified method for using PDFA in the context of transition metal-mediated [^18^F]trifluoromethylthiolation of α-bromo carbonyl compounds [66]. These new studies gave a clearer picture of the mechanism associated with the synthesis pathway. This rapid method afforded compounds at the low molar activity of 0.074 GBq/μmol.

**Figure 11:**
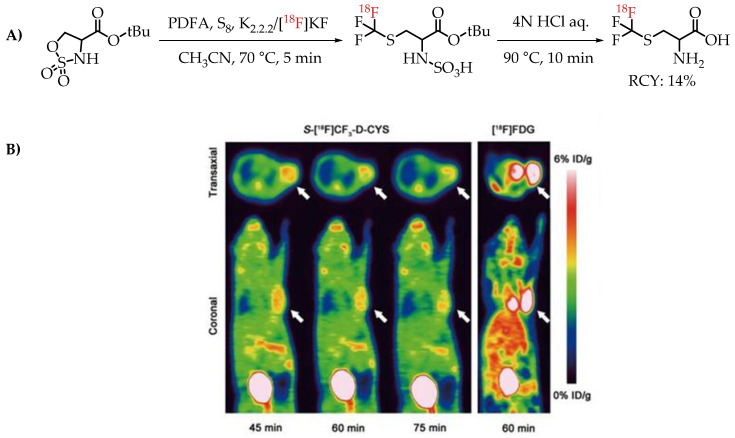
(**A**) [^18^F]Trifluoromethyl cysteine synthesis from cyclic sulfamidates. (**B**) Static PET images of C6 glioma-bearing mice scanned at 45, 60 and 75 min after injection of *S*-[^18^F]CF_3_ D CYS and 60 min after injection of [^18^F]FDG (the white arrow indicates the tumour); (adopted from [67]; (RCY: radiochemical yield).

[^18^F]Trifluoromethyl cysteine as an amino acid mimicking radiotracer for imaging glioma was explored by Tang et al. [67]. The [^18^F]trifluoromethylthiolation reaction was achieved by employing PDFA and S_8_, on cyclic sulfamidates. The one-step assembly of [^18^F]trifluoromethyl cysteine represents an interesting method for the synthesis of the fluorine-18-labelled amino acid mimics that was tested for PET imaging in C6 glioma-bearing mice (Figure 11).

**Scheme 18:**
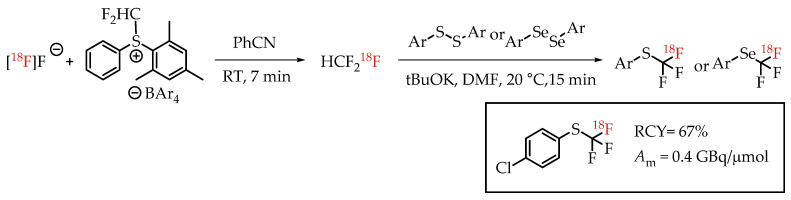
[^18^F]Trifluoromethylthiolation and [^18^F]trifluoromethylselenation via [^18^F]Fluoroform. (RCY: radiochemical yield; A_m_: molar activity.)

Jubault et al. reported a method for [^18^F]trifluoromethylthiolation and [^18^F]trifluoromethyl-selenation reactions via [^18^F]fluoroform (Scheme 18) [68]. [^18^F]fluoroform was generated by a procedure from the same group [57]. [^18^F]fluoroform was trapped by an aromatic disulfide (or diselenide) and a base. This reaction provided the corresponding [^18^F]trifluoromethyl-sulfur/selenium compounds in low molar activity of 0.38 GBq/μmol.

**Scheme 19:**
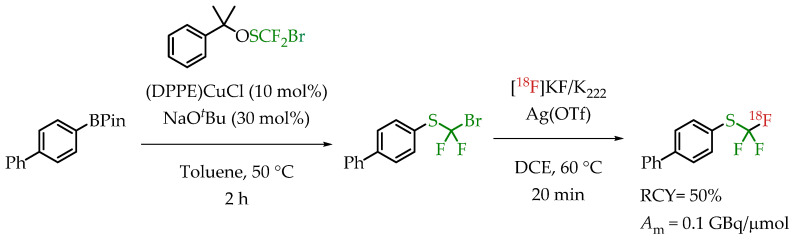
α-Cumyl bromodifluoromethanesulfenate-mediated [^18^F]trifluoromethylthiolation of boronate esters. (RCY: radiochemical yield; A_m_: molar activity.)

A novel reagent for bromodifluoromethylthiolation reactions was reported by Wu et al. [69]. α-Cumyl bromodifluoro-methanesulfenate was used for transferring a bromodifluoromethylthio group onto an aryl boronic ester (Scheme 19). Subsequent radiofluorination with n.c.a. [^18^F]fluoride in the presence of AgOTf gave compounds containing a [^18^F]CF_3_-S group in good radiochemical yields but a low molar activity in the range of 0.1 GBq/μmol.

## 5. Summary and Conclusions

[^18^F]Trifluoromethylation chemistry is a rapidly evolving area in radiopharmaceutical sciences for PET imaging. Considered a challenging and exotic subset of ^18^F radiofluorination reactions in the past, [^18^F]trifluoromethylations are now becoming a more established radiofluorination technique in radiopharmaceutical chemistry. The increasing presence of CF_3_ groups in many drug molecules and the continuous growth of PET as a cutting-edge functional imaging technique in biomedical and clinical research are the primary drivers for developing novel [^18^F]trifluoromethylation chemistry strategies. In comparison with traditional radiofluorinations, there are unique challenges associated with [^18^F]trifluoromethylations. A major challenge is the low molar activity of <5 GBq/μmol of many [^18^F]CF_3_ group-containing PET radiotracers. Traditional radiofluorination methodologies with n.c.a. [^18^F]fluoride typically achieve high molar activities >40 GBq/μmol. There is only a handful of reported [^18^F]trifluoromethylation methods that can achieve that. The low molar activity challenge of many [^18^F]CF_3_ group-containing PET radiotracers is observed with the majority of the reported methods. However, some of the discussed four major synthesis strategies for the installation of a [^18^F]CF_3_ group could offer opportunities to obtain radiotracers at a high molar activity.

One-pot isotopic exchange reactions are quite limited when it comes to producing radiotracers at higher molar activities due to the inherent challenge of removing the chemically and physically identical labelling precursor. A two-step isotopic exchange radiofluorination involving an isolable metal complex yielded the radiotracer at a low molar activity of 0.3 GBq/μmol. Nucleophilic [^18^F]trifluoromethylation chemistry starting from n.c.a [^18^F]fluoride is the most widely explored and successful method for [^18^F]trifluoromethylation reactions. However, the synthesis of difluoromethyl groups with a suitable leaving group can pose a special synthetic challenge associated with this strategy. Both aliphatic and aromatic nucleophilic [^18^F]trifluoromethylation reactions provide radiotracers in reasonable radiochemical yields and high molar activities in the range of 30–120 GBq/μmol. The obtained favourable radiochemical yields and high molar activities predestine nucleophilic [^18^F]trifluoromethylation reactions as a method of choice for the preparation of [^18^F]CF_3_ group-containing radiotracers suitable for PET imaging experiments.

Difluorocarbene-based [^18^F]trifluoromethylation strategies have also rapidly evolved due to recent advances in designing and synthesising suitable difluorocarbene precursors. [^18^F]Trifluoromethylation reactions with difluorocarbenes always require the presence of a transition metal, mainly copper. Undesired side reactions such as defluorination or elimination of [^18^F]fluoroform are also possible with this method. Radiolabelling strategies with [^18^F]fluoroform as a [^18^F]trifluoro-methylating agent was also explored. Despite the operational complexity of [^18^F]fluoroform-based synthesis strategies, the resulting radiotracers were generally obtained in good radiochemical yields and moderate to high molar activities. Electrophilic [^18^F]fluorinating reagents are not as widely used and tested as to their nucleophilic counterparts for [^18^F]trifluoromethylations. Recent utilization of reagents such as [^18^F]Selectfluor that are capable of n.c.a. radiofluorinations offers an opportunity for improving the molar activity of the obtained radiotracers. Several [^18^F]trifluoromethyl transfer reagents such as [^18^F]Umemoto reagents are also significant compounds used for electrophilic [^18^F]trifluoromethylations. Alkyl trifluoromethyl labelling strategies with electrophilic [^18^F]fluorinating reagents proceed with moderate radiochemical yields and low molar activities. The increasing number of drugs containing trifluoromethoxy (CF_3_O-) and trifluoromethylthio (CF_3_S-) groups also require innovative radiochemistry for the synthesis of respective fluorine-18-labelled radiotracers. The increasing number of innovative [^18^F]trifluoromethylation chemistries is becoming a significant driving force for the development of novel fluorine-18-labelled imaging agents. Although the reports discussed in this review demonstrate the considerable progress in [^18^F]trifluoromethylation chemistry over the last decade, fluorine-18 radiochemistry is still heavily dominated by synthesis strategies yielding mono-radiofluorinated radiotracers (aryl-^18^F and alkyl-^18^F) by using traditional nucleophilic and electrophilic radiofluorinations. Thus, further investments in the development of novel [^18^F]trifluoromethylation chemistry are required. The development of innovative [^18^F]trifluoromethylation chemistry will significantly impact future radiopharmaceutical chemistry and preclinical and clinical molecular imaging.

## Data Availability

Not applicable.
